# Efficacy and Safety of Hirudotherapy for Improving Sperm Quality Parameters in Male Infertility: A Randomized Controlled Trial

**DOI:** 10.1002/hsr2.71835

**Published:** 2026-02-23

**Authors:** Mozhde‐Sadat Abtahi‐Forooshani, Shahla Roozbehani, Mahnoosh Fatemi, Ali Noori

**Affiliations:** ^1^ Department of Biology, Fal.C. Islamic Azad University Isfahan Iran

**Keywords:** infertility, leech saliva, leech therapy, sperm function

## Abstract

**Background and Aims:**

Male infertility, often characterized by impaired sperm parameters, remains a significant clinical challenge. This study aimed to evaluate the efficacy and safety of hirudotherapy (medicinal leech therapy) in improving sperm quality parameters in men with idiopathic infertility.

**Methods:**

In this randomized controlled trial, 50 male volunteers aged 20–50 years were randomly assigned to either an experimental group receiving weekly leech therapy for 3 months (*n* = 25) or a control group receiving no intervention (*n* = 25). Semen parameters were assessed at baseline and after the intervention period. Evaluations included semen analysis according to WHO guidelines, sperm DNA fragmentation by TUNEL assay, protamine deficiency by chromomycin A3 staining, and assessments of sperm viability and morphology using flow cytometry and Diff‐Quik staining. Statistical analyses were performed using SPSS version 21.0, with significance set at *p* < 0.05.

**Results:**

Compared to the control group, leech therapy significantly improved several sperm quality parameters. Sperm concentration increased from 57.7 ± 17.1 to 63.4 ± 13.1 million/mL (*p* = 0.006). Total sperm count, progressive motility, and normal morphology also improved significantly (*p* < 0.05). DNA fragmentation decreased significantly in the experimental group compared to controls (mean difference: −3.9%; *p* = 0.04). Protamine deficiency also showed a significant reduction (*p* = 0.02). No adverse effects related to the intervention were observed.

**Conclusion:**

This randomized controlled trial suggests that hirudotherapy may improve sperm quality parameters, including concentration, motility, morphology, and DNA integrity, in men with idiopathic infertility. These findings support further investigation of leech therapy as a complementary approach to male infertility management.

**Trial Registration:** IRCT20230502058045N.

**Abbreviations**
ART: assisted reproductive technologies
ICSI: intracytoplasmic sperm injection
IUI: including intrauterine insemination
IVF: in vitro fertilization
TESE: testicular sperm extraction

## 1 Introduction

Male infertility, defined as the inability to conceive with a fertile female partner after 12 months of regular unprotected intercourse, is a multifactorial condition with various etiologies [1]. Key physiological issues include impaired sperm production, transport, or both, manifesting as reduced sperm count (oligozoospermia), motility (asthenozoospermia), abnormal morphology (teratozoospermia), or azoospermia [2]. Hormonal disorders, genetic defects, anatomical obstructions, and immunological factors may also contribute [3]. Globally, around 7% of men are affected, representing a notable portion of infertility cases [4].

Environmental pollutants, lifestyle‐related disorders, and delayed parenthood are among the drivers of rising male infertility rates [5]. Psychological stress and the high cost of assisted reproductive technologies (ART) underscore the need for improved diagnostic and therapeutic strategies [6]. A multidisciplinary approach combining research, clinical practice, and public health is necessary to mitigate its impact [7].

Current treatments include lifestyle modification, hormonal therapy, ART (e.g., IUI, IVF‐ICSI), and microsurgery (e.g., varicocelectomy, TESE) [8]. However, these methods often have limited success, high costs, and fail to address underlying causes [8]. This highlights the demand for novel options such as regenerative medicine, gene therapy, and alternative approaches like leech therapy [9].

Leech therapy (hirudotherapy) involves applying medicinal leeches to improve circulation and reduce inflammation [10]. In male infertility, it is proposed to enhance blood flow to reproductive organs and potentially improve sperm parameters [11]. Bioactive substances in leech saliva, such as hirudin, may aid in this process [10, 11]. However, scientific evidence is limited and mixed, with concerns about infection, allergic reactions, and lack of standardized protocols [12].

While traditional medicine supports its use, rigorous clinical data are lacking [10, 11, 12]. This study aims to assess the effects of leech therapy on sperm quality through controlled evaluation of semen parameters pre‐ and posttreatment. The findings will help determine its therapeutic potential and guide future evidence‐based applications.

## 2 Methods

### 2.1 Study Population

This study was approved by the ethics committee of Islamic Azad University, Falavarjan Branch (IR.IAU.FALA.REC.1402.015) and registered under Code 172548642186552946021162776866. This study was also registered with the clinical trial ID IRCT20230502058045N1 on the website irct.behdasht.gov.ir/user/trial/. Participants with idiopathic male infertility were recruited from the Isfahan Royan Andrology Center based on WHO semen criteria, clinical history, and exclusion of known causes. Informed consent was obtained. Inclusion: men aged 20–50. Exclusion: urinary symptoms, sexual dysfunction, bleeding disorders, cognitive impairment, or participation in other trials.

### 2.2 Randomization and Masking

**Figure 1:**
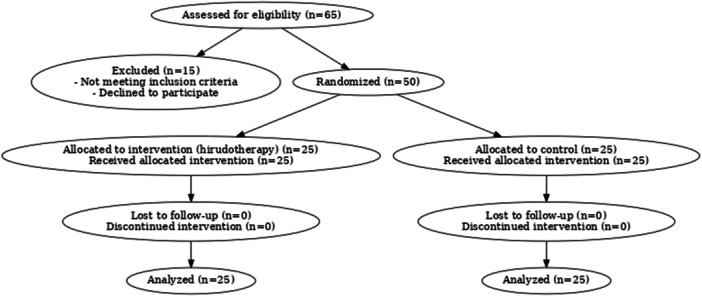
CONSORT flow diagram showing the progress of participants through the phases of the randomized controlled trial of hirudotherapy for male infertility, including enrollment, allocation, follow‐up, and analysis.

Fifty men were randomized into control and experimental groups (*n* = 25 each) using computer‐generated sequences and sealed envelopes. Blinding was not feasible. Both groups underwent baseline semen analysis; only the experimental group received leech therapy. Posttreatment analyses were done after 3 months (Figure 1).

### 2.3 Leech Therapy Protocol

Certified Hirudo medicinalis leeches were applied weekly for 12 weeks (2–3/session), targeting the inguinal and lower abdominal regions. Leech application followed standardized protocols (AYUSH, CCRUM), with skin disinfection and consistent treatment conditions. Each session lasted 30–45 min. Used leeches were disposed of ethically. Blood loss (~10–15 mL/session) was monitored; no significant hemoglobin changes occurred.

### 2.4 Semen Sample Collection

Samples were collected after 3–4 days of abstinence and analyzed within 1 h. WHO criteria were used to assess sperm concentration, motility, and morphology. Additional tests included DNA fragmentation (TUNEL), chromatin integrity (CMA3), and oxidative stress (H2DCFDA).

### 2.5 Sperm Quality and Functional Assessments

- Count and motility: Assessed via Makler chamber and CASA.
- Morphology: Evaluated using Papanicolaou and Diff‐Quik staining.
- DNA fragmentation: Measured via TUNEL assay and fluorescence microscopy.
- Chromatin integrity: Assessed with CMA3 staining under fluorescent microscopy.
- Oxidative stress: Quantified using H2DCFDA and flow cytometry.
- SCSA: Performed via Acridine Orange staining and flow cytometry.

### 2.6 Statistical Analysis

Primary outcome: sperm concentration. Secondary outcomes: motility, morphology, viability, DNA integrity, and patient satisfaction. Data were analyzed using SPSS v21 with parametric and nonparametric tests (*T*‐test, Mann–Whitney, Wilcoxon, McNemar, ANCOVA). Significance set at *p* < 0.05. The study followed SAMPL reporting guidelines.

## 3 Results

### 3.1 Participant Flow

We enlisted 50 men from January to April 2024. Participants were randomly allocated to treatment and control groups, each consisting of 25 individuals. The loss to follow‐up rate was similar for both groups (0% in the treatment group and 0% in the control group), and no males left the trial. Table 1 presents the ages of participants categorized into experimental and control groups. The predominant age group among participants in the control groups was 30–40 years, although the experimental groups also included individuals aged 30–40 (Table 1).

**Table 1:** Characterization of the age distribution of the participants in this study, divided into experimental and control groups.

Groups	Age scale	The profile of the perpetrators			
		Number	Percentage	Cumulative percentage	Mean ± standard deviation
Experimental group	≤ 30	7	23.3%	23.3%	35.5 ± 7.1
	30 ≤ *X* ≤ 40	19	63.4%	86.7%	
	≥ 40	4	13.3%	100%	
Control group	≤ 30	1	3.3%	3.3%	37.9 ± 4.7
	30 ≤ *X* ≤ 40	22	73.4%	76.7%	
	≥ 40	7	23.3%	100%	

### 3.2 Baseline Characteristics

During the initial semen collection, baseline data were comparable, and there was no variation in the technique or timing of collection prior to analysis. The study of pre‐therapy semen from the two control and experimental groups revealed no significant difference between them (Table 1).

### 3.3 Semen Quality

Pretreatment assessment showed that most participants in both the control and experimental groups had semen pH around 7, within the normal range of 7.2–7.8, with a few exhibiting pH of 8, which may indicate infection. White blood cell (WBC) counts in semen were also largely normal (0–1 WBC per high‐power field), indicating that the majority of participants did not have leukocytospermia. Table 2 indicates that most control and experimental group participants had a WBC count of one. Hence, the WBC concentration in the seminal fluid of most participants was within the normal range.

**Table 2:** Description of the characteristic pH level and white blood cells of seminal fluid in the studied population.

Groups	pH range	Number	Percentage	Mean ± standard deviation
*pH level of seminal fluid*				
Experimental group (*n* = 25)	pH = 7	16	64%	12.5 ± 3.5
	pH = 8	9	36%	
Control group (*n* = 25)	pH = 7	18	72%	12.5 ± 5.5
	pH = 8	7	28%	
*Seminal fluid white blood cells*				
Experimental group (*n* = 25)	hpf = 1	20	80%	8.33 ± 8.33
	hpf = 2	4	16%	
	hpf > 3	1	4%	
Control group (*n* = 25)	hpf = 1	19	76%	8.33 ± 7.17
	hpf = 2	5	20%	
	hpf > 3	1	4%	

### 3.4 Primary Outcome of Pre‐Therapy Semen Analysis

Pre‐therapy comparisons showed no significant differences between the control and experimental groups for most sperm parameters, including abstinence days, sperm concentration, semen volume, total sperm count, motility, immobile sperm, head and neck defects, immature cells, and DNA fragmentation (Table 3). Significant differences were observed for abnormal morphology (experimental: 96.7 ± 1.4 vs. control: 95.9 ± 1.4, *p* = 0.0299), tail defects (experimental: 28.7 ± 3.8 vs. control: 27.5 ± 4.3, *p* = 0.0330), and protamine levels (experimental: 22.3 ± 6.9 vs. control: 21.5 ± 6.6, *p* = 0.0299) (Table 3).

**Table 3:** Comparison of baseline features, conventional semen parameters, and sperm DNA fragmentation levels between samples obtained from the experimental and control groups before leech treatment (pre‐therapy).

Baseline characteristics of pre‐therapy			
Baseline	Control group (*n* = 25)	Experimental group (*n* = 25)	*p*
Age of men (average years)	37.9	35.5	0.0035
Days of abstinence	3–4	3–4	*N*s (> 0.9999)
Collection to analysis (hours)	1.0 (1.0–1.5)	1.0 (1.0–1.0)	0.0241
*Semen parameters*			
Sperm concentration (×10^6^/mL)	57.4 ± 18.8	57.7 ± 15.1	*N*s (*p* = 0.5714)
Semen volume (mL)	3.9	3.5	*N*s (*p* = 0.1172)
Total sperm count (×10^6^)	216.4 ± 19.2	214.9 ± 14.3	*N*s (*p* = 0.2039)
Progressive motility (%)	35.7 ± 10.8	36.1 ± 9.7	*N*s (*p* = 0.2155)
Nonprogressive mobility (%)	20.3 ± 3.8	20.1 ± 3.7	*N*s (*p* = 0.2929)
Full mobility (%)	60.9 ± 13.1	61.2 ± 10.4	*N*s (*p* = 0.3118)
Immobile sperm (%)	39.1 ± 13.1	38.8 ± 9.9	*N*s (*p* = 0.1679)
Normal morphology (%)	4.1 ± 1.4	3.2 ± 1.4	*N*s (*p* = 0.0565)
Abnormal morphology (%)	95.9 ± 1.4	96.7 ± 1.4	0.0299
Head defect (%)	95.4 ± 1.9	96.1 ± 1.4	*N*s (*p* = 0.2317)
Neck defect (%)	32.7 ± 4.1	32.4 ± 3.5	*N*s (*p* = 0.1679)
Tail defect (%)	27.5 ± 4.3	28.7 ± 3.8	0.0330
Immature cells (%)	2.9 ± 1.1	2.8 ± 1.1	*N*s (*p* = 0.5528)
Protamine level (%)	21.5 ± 6.6	22.3 ± 6.9	0.0299
DNA fragmentation (%)	18.5 ± 7.8	18.1 ± 8.1	*N*s (*p* = 0.1056)

### 3.5 Secondary Outcomes of Post‐Therapy Semen Analysis

Leech treatment was administered to the patients in the experimental group for 3 months. An illustration of leech treatment is included in Supporting Materials (Figure S1). The ITT analysis revealed a significant difference in semen volume, total sperm count, percentage of progressive motility, percentage of normal morphology, sperm viability, and percentage of sperm with fragmented DNA in the experimental group. The sperm concentrations in the control and experimental groups were 57.7 ± 17.1 and 63.4 ± 13.1, respectively (*p* = 0.0061). The experimental and control groups had full mobility of 65.2 ± 8.5 and 56.7 ± 13.1, respectively (*p* = 0.0011). The control and experimental groups had normal morphology of 4.1 ± 1.4 and 5.7 ± 1.8, respectively (*p* = 0.0190). The control and experimental groups had DNA fragmentation rates of 19.1 ± 8.1 and 15.1 ± 6.7, respectively (*p* = 0.0012). According to the test findings, there is a significant difference (*p* < 0.05) between the means of the experimental and control groups in sperm parameters. Consequently, it may be inferred that the groups exhibited a statistically significant difference. There was a notable disparity between the experimental and control groups for sperm parameters, even accounting for the influence of pre‐therapy values. This signifies the rejection of the null hypothesis and the validation of the hypothesis being examined, predicated on the impact of leech treatment on enhancing sperm parameters (Table 4).

**Table 4:** Comparison of baseline characteristics, conventional semen parameters, and sperm DNA fragmentation levels between samples obtained from the experimental and control groups among persons after leech treatment.

Baseline characteristics of post‐therapy (leech therapy)			
Baseline	Control group (*n* = 25)	Experimental group (*n* = 25)	*p*
Age of men (average years)	37.9	35.5	0.0035
Days of abstinence	3–4	3–4	*N*s (> 0.9999)
Collection to analysis (hours)	1.0 (1.0–1.5)	1.0 (1.0–1.0)	0.0241
*Semen parameters*			
Sperm concentration (×10^6^/mL)	57.7 ± 17.1	63.4 ± 13.1	0.0061
Semen volume (mL)	4.1	3.9	*N*s (*p* = 0.2929)
Total sperm count (×10^6^)	228.5 ± 21.9	248.8 ± 18.1	0.0010
Progressive motility (%)	36.2 ± 11.9	46.1 ± 8.8	0.0005
Nonprogressive mobility (%)	20.8 ± 1.4	19.1 ± 3.5	*N*s (*p* = 0.054)
Full mobility (%)	56.7 ± 13.1	65.2 ± 8.5	0.0011
Immobile sperm (%)	43.3 ± 13.1	34.9 ± 8.7	0.0018
Normal morphology (%)	4.1 ± 1.4	5.7 ± 1.8	0.0190
Abnormal morphology (%)	95.9 ± 1.4	94.2 ± 1.8	0.0169
Head defect (%)	95.7 ± 1.3	94.3 ± 1.8	0.0101
Neck defect (%)	32.4 ± 1.4	30.1 ± 4.4	0.0307
Tail defect (%)	27.1 ± 4.8	25.2 ± 1.4	0.0136
Immature cells (%)	2.7 ± 1.1	1.3 ± 0.85	0.0246
Protamine level (%)	21.7 ± 6.9	19.6 ± 7.1	0.0177
DNA fragmentation (%)	19.1 ± 8.1	15.1 ± 6.7	0.0012

Subsequently, a covariance analysis was conducted to ascertain the disparity in protamine levels across the groups, finalizing the interpretation of the descriptive data. Table 6 indicates that at a significance level of 0.02, the mean protamine levels in the pre‐therapy and post‐therapy groups vary. Nonetheless, the eta coefficient indicates that this difference is insignificant (Table 5).

**Table 5:** Covariance analysis to ascertain the disparity in protamine levels among groups.

Resources		Sum of squares	Degree of freedom	Mean square	*F*	Level of significance	Parabolic eta squared
Protamine level	Modified model	1737.4	2	868.7	41.1	*p* < 0.001	0.59
	Width from the origin	62.4	1	62.4	2.9	*p* = 0.09	0.04
	Pretest	1670.1	1	1670.1	78.9	*p* < 0.001	0.58
	Groups	112.8	1	112.8	5.3	*p* = 0.02	0.08
	Error	1205	57	21.1	—	—	—
	Total	28,652.2	60	—	—	—	—

The TUNEL test and the Sperm Chromatin Structure test (SCSA) are distinct techniques for evaluating DNA damage in spermatozoa. The TUNEL test has superior accuracy and detection capabilities in recognizing genuine DNA damage, especially in the analysis of double‐strand breaks. This test effectively evaluates the degree of DNA damage. The SCSA test utilizes flow cytometry, enabling the analysis of a substantial quantity of sperm in a reduced timeframe while thoroughly evaluating chromatin integrity. The outcomes of this investigation were assessed using covariance analysis and independent *t*‐tests. Table 6 presents the results of the *F* test conducted to assess the effects between participants, which investigated the study hypothesis about the efficacy of leech treatment in mitigating sperm nuclear DNA damage. The test findings indicated a statistically significant difference in the average sperm nuclear DNA damage between the experimental and control groups (*p* < 0.05). Consequently, the groups exhibited a statistically significant difference from one another. A notable disparity existed between the experimental and control groups post‐leech treatment for sperm nuclear DNA damage (*p* = 0.00), with pre‐therapy averages being reduced. This validated the beneficial impact of leech treatment on reducing sperm nuclear DNA damage. Table 6 presents the corrected mean of sperm parameters after treatment (post‐leech therapy) for the control and experimental groups. This table indicates that the influence of the pretreatment variable (before leech therapy) has been statistically eradicated.

**Table 6:** The impact of leech treatment in reducing sperm nuclear DNA damage varies amongst subjects.

Resources		Sum of squares	Degree of freedom	Mean square	*F*	Level of significance	Parabolic eta squared
Protamine level	Modified model	345.7	2	172.2	3.2	*p* = 0.04	0.10
	Width from the origin	1806.8	1	1806.8	33.7	*p* < 0.001	0.37
	Pretest	106.1	1	106.1	1.9	*p* = 0.16	0.03
	Groups	231.7	1	231.7	4.3	*p* = 0.04	0.07
	Error	3056.1	57	53.6	—	—	—
	Total	20,813.4	60	—	—	—	—

The findings in Table 7 (as indicated by the mean difference column) demonstrate that the scores for the variables concentration, total sperm count, progressive motility, nonprogressive motility, complete motility of immobile sperm, normal morphology, abnormal morphology, head defect, leech therapy resulting in neck defect, reduction of tail defect, and immature cells exhibited significant differences between the experimental groups and the control, which was statistically validated (*p* < 0.05).

**Table 7:** After leech therapy, the means of posttest variables for the control and experimental groups were adjusted.

Dependent variable	Group comparison	Mean difference	Standard deviation	Significant value	95% confidence interval	
					Lower limit	Upper limit
Sperm concentration	Experimental–control	5.8	1.8	0.04	0.18	11.4
Total sperm count	Experimental–control	37.9	18.8	0.04	0.23	75.6
Progressive motility	Experimental–control	8.4	2.2	0.00	3.9	12.9
Nonprogressive mobility	Experimental–control	−2.1	1.1	0.04	−4.2	−0.02
Full mobility	Experimental–control	5.1	2.1	0.01	0.91	9.2
Immobile sperm	Experimental–control	−4.2	2.1	0.04	−8.3	−0.21
Normal morphology	Experimental–control	1.7	0.44	0.00	0.85	2.5
Abnormal morphology	Experimental–control	−1.7	0.44	0.00	−2.5	−0.84
Head defect	Experimental–control	−1.5	0.41	0.00	−2.3	−0.71
Neck defect	Experimental–control	−2.1	1.1	0.04	−4.3	−0.04
Tail defect	Experimental–control	−2.3	1.1	0.03	−4.5	−0.22
Immature cells	Experimental–control	−0.72	0.23	0.01	−1.1	−0.24

The results of the TUNEL and SCSA tests assessing sperm DNA fragmentation are presented in the Supporting Materials under Tables S1 and S2. These tables detail the pre‐ and posttreatment comparisons between the control and experimental groups.

## 4 Discussion

### 4.1 Main Findings

This study demonstrates that leech therapy can significantly improve sperm parameters, including concentration, motility, morphology, and chromatin integrity [13]. Posttreatment analysis revealed a marked increase in sperm concentration, consistent with previous findings [13]. Leech saliva contains bioactive compounds with anti‐inflammatory and antioxidant properties that may enhance testicular blood flow, stimulate Leydig cell activity, and increase testosterone levels—key factors in spermatogenesis [14]. Additionally, the therapy's stress‐reducing effects may contribute to improved sperm production [13, 14].

Leech therapy also led to improvements in progressive motility, with a decline in nonprogressive and immotile sperm [13, 15]. Enhanced circulation and oxygen delivery to the testes, driven by enzymes like hirudin, support mitochondrial function and ATP production, vital for sperm motility [13, 15]. These effects may counter oxidative damage that compromises flagellar integrity [13, 15], while antioxidants in leech saliva help neutralize ROS, thereby improving overall motility [13, 15].

A reduction in immotile sperm further supports the hypothesis that leech therapy relieves testicular inflammation and improves the microvascular environment, preventing toxin accumulation [16]. These physiological changes foster a biochemical environment that supports functional germ cell development.

In terms of sperm morphology, the treatment increased the percentage of normally shaped sperm while reducing head, neck, and tail abnormalities, as well as immature cells [17, 18]. This improvement likely results from the detoxifying effects of leech therapy, which enhance cellular metabolism and reduce oxidative insults [13, 17]. Past research also supports the notion that improved circulation and reduced oxidative stress help correct morphological defects [19, 20].

Importantly, leech therapy significantly enhanced protamine levels in sperm, which correlates with better chromatin packaging and DNA integrity [13, 21]. Protamines stabilize the sperm nucleus and prevent DNA fragmentation, which is crucial for fertility. Anti‐inflammatory enzymes from leech saliva may reduce chronic inflammation, creating favorable conditions for sperm development [13, 22, 23]. Improved testicular circulation may also facilitate nutrient delivery and support spermatogenic cell function, leading to increased protamine incorporation [13, 22].

Finally, TUNEL and SCSA assays confirmed a significant reduction in sperm DNA fragmentation posttreatment, consistent with prior evidence [13, 24]. This reduction likely results from enhanced protamine‐mediated chromatin stability and decreased oxidative stress. The findings suggest that leech therapy, by improving blood flow, reducing inflammation, and enhancing chromatin integrity, may offer a viable adjunctive treatment for male infertility [13, 24, 25].

### 4.2 Strengths of Our Study

A major strength of our study is its randomized controlled trial design, which enhances the internal validity and reduces selection bias. By including both intention‐to‐treat and per‐protocol analyses, we ensured a robust evaluation of the intervention's true effect. Comprehensive sperm quality assessment was performed using multiple complementary methods, including WHO standard semen analysis, DNA fragmentation by TUNEL assay, protamine deficiency staining, and advanced morphology and viability testing, providing a thorough and multidimensional evaluation of treatment impact. Furthermore, our study is among the first to systematically investigate the effects of hirudotherapy on male fertility parameters, addressing an important but underexplored area in reproductive medicine.

### 4.3 Limitations of Our Study

This study has some limitations that should be considered. The sample size was relatively small, which may limit the generalizability of the findings to broader populations. The intervention period was limited to 3 months, so longer‐term effects and the sustainability of the improvements remain unclear. Additionally, the study lacked blinding due to the nature of the intervention, which may introduce some degree of observer bias. Finally, although multiple sperm parameters were assessed, we did not include live birth or pregnancy outcomes, which would provide direct evidence of clinical relevance.

## 5 Conclusions

This research shows that leech treatment may positively influence sperm parameters, functional capacity, protamine levels, and DNA integrity. Leech treatment may positively influence male reproductive health by enhancing normal sperm morphology, diminishing abnormalities in the head, neck, and tail, and decreasing the number of immature cells. Nevertheless, further research is required to definitively establish the efficacy of leech treatment in enhancing sperm quality and augmenting the likelihood of conception.

## Author Contributions

**Mozhde‐Sadat Abtahi‐Forooshani:** writing – original draft, software, project administration, funding acquisition. **Shahla Roozbehani:** methodology, writing – original draft. **Mahnoosh Fatemi:** investigation, writing – original draft. **Ali Noori:** writing – review and editing, visualization, validation, resources, supervision. All authors have read and approved the final version of the manuscript.

## Funding

The authors received no specific funding for this work.

## Disclosure

The lead author Shahla Roozbehani affirms that this manuscript is an honest, accurate, and transparent account of the study being reported; that no important aspects of the study have been omitted; and that any discrepancies from the study as planned (and, if relevant, registered) have been explained. Shahla Roozbehani had full access to all of the data in this study and takes complete responsibility for the integrity of the data and the accuracy of the data analysis.

## Ethics Statement

The research received approval from the institutional reviewing board of the Research Ethics Committees at Islamic Azad University, Falavarjan branch (Approval Number: IR.IAU.FALA.REC.1402.015) and was registered with the Isfahan Royan Clinical Trial Registrar (http://isfahan.royan.org/). This study was approved by the ethics committee of Islamic Azad University, Falavarjan Branch (IR.IAU.FALA.REC.1402.015) and registered under Code 172548642186552946021162776866. This study was also registered with the clinical trial ID IRCT20230502058045N1 on the website irct.behdasht.gov.ir/user/trial/.

## Conflicts of Interest

The authors declare no conflicts of interest.

## Supporting information


**Figure S1:** Leech therapy was performed on the experimental group patients.
**Table S1:** The adjusted post‐test averages for the control and experimental groups following leech treatment.
**Table S2:** The *T*‐test was used to determine if there was a significant difference in the quantity of DNA damage in the experimental group.

## Data Availability

The data utilized in this study are available upon reasonable request from the corresponding author.

## References

[1] E. Babakhanzadeh , M. Nazari , S. Ghasemifar , and A. Khodadadian , “Some of the Factors Involved in Male Infertility: A Prospective Review,” International Journal of General Medicine no. 13 (February 2020): 29–41. 32104049 10.2147/IJGM.S241099 PMC7008178

[2] N. Gatimel , J. Moreau , J. Parinaud , and R. D. Léandri , “Sperm Morphology: Assessment, Pathophysiology, Clinical Relevance, and State of the Art in 2017,” Andrology 5, no. 5 (September 2017): 845–862. 28692759 10.1111/andr.12389

[3] A. W. Michels and G. S. Eisenbarth , “Immunologic Endocrine Disorders,” Journal of Allergy and Clinical Immunology 125, no. 2 (February 2010): S226–S237. 20176260 10.1016/j.jaci.2009.09.053 PMC2835296

[4] B. Huang , Z. Wang , Y. Kong , M. Jin , and L. Ma , “Global, Regional and National Burden of Male Infertility in 204 Countries and Territories Between 1990 and 2019: An Analysis of Global Burden of Disease Study,” BMC Public Health 23, no. 1 (November 2023): 2195. 37940907 10.1186/s12889-023-16793-3 PMC10631182

[5] R. Bala , V. Singh , S. Rajender , and K. Singh , “Environment, Lifestyle, and Female Infertility,” Reproductive Sciences 28, no. 3 (March 2021): 617–638. 32748224 10.1007/s43032-020-00279-3

[6] M. P. Connolly , S. Hoorens , and G. M. Chambers , “The Costs and Consequences of Assisted Reproductive Technology: An Economic Perspective,” Human Reproduction Update 16, no. 6 (November 2010): 603–613. 20530804 10.1093/humupd/dmq013

[7] F. D. Yahya , “The Role of Multidisciplinary Approaches in Public Health Research: A Literature Review,” Advances in Healthcare Research 1, no. 2 (August 2023): 55–62.

[8] A. Kaltsas , F. Dimitriadis , D. Zachariou , et al., “From Diagnosis to Treatment: Comprehensive Care by Reproductive Urologists in Assisted Reproductive Technology,” Medicina 59, no. 10 (October 2023): 1835. 37893553 10.3390/medicina59101835 PMC10608107

[9] M. Rasekh , M. S. Arshad , and Z. Ahmad , “Advances in Drug Delivery Integrated With Regenerative Medicine: Innovations, Challenges, and Future Frontiers,” Pharmaceutics 17, no. 4 (April 2025): 456. 40284451 10.3390/pharmaceutics17040456 PMC12030587

[10] S. Abdullah , L. Dar , A. Rashid , and A. Tewari , “Hirudotherapy/Leech Therapy: Applications and Indications in Surgery,” Archives of Clinical and Experimental Surgery (ACES) 1, no. 3 (January 2012): 172–180.

[11] A. Majzoub and A. Agarwal , “Systematic Review of Antioxidant Types and Doses in Male Infertility: Benefits on Semen Parameters, Advanced Sperm Function, Assisted Reproduction and Live‐Birth Rate,” Arab Journal of Urology 16, no. 1 (March 2018): 113–124. 29713542 10.1016/j.aju.2017.11.013 PMC5922223

[12] M. Pourrahimi , M. Abdi , and R. Ghods , “Complications of Leech Therapy,” Avicenna Journal of Phytomedicine 10, no. 3 (May 2020): 222–234. 32523877 PMC7256282

[13] F. Davoodi , S. Taheri , A. Raisi , et al., “Leech Therapy (*Hirudo medicinalis*) Attenuates Testicular Damages Induced by Testicular Ischemia/Reperfusion in an Animal Model,” BMC Veterinary Research 17, no. 1 (July 2021): 256. 34315461 10.1186/s12917-021-02951-5 PMC8314469

[14] B. Bakhshinejad , M. Karimi , and M. Sadeghizadeh , “Bacteriophages and Medical Oncology: Targeted Gene Therapy of Cancer,” Medical Oncology 31, no. 8 (August 2014): 110. 25012686 10.1007/s12032-014-0110-9

[15] E. Evgeni and P. Kothari , “Sperm Motility,” in Human Semen Analysis: From the WHO Manual to the Clinical Management of Infertile Men (Springer International Publishing, 2024), 61–101.

[16] S. Wu , Y. Zhou , Y. Wang , and Z. Zhang , “Therapeutic Potentials of Medicinal Leech in Chinese Medicine,” American Journal of Chinese Medicine 52, no. 04 (June 2024): 1027–1051. 38879745 10.1142/S0192415X24500423

[17] H. E. Chemes and C. Alvarez Sedo , “Tales of the Tail and Sperm Head Aches Changing Concepts on the Prognostic Significance of Sperm Pathologies Affecting the Head, Neck and Tail,” Asian Journal of Andrology 14, no. 1 (December 2011): 14–23. 22198630 10.1038/aja.2011.168 PMC3735144

[18] T. Piri‐Gharaghie , G. Ghajari , G. Rezaeizadeh , M. Adil , and M. H. Mahdi , “A Novel Vaccine Strategy Against Brucellosis Using *Brucella abortus* Multi‐Epitope OMPs Vaccine Based on *Lactococcus lactis* Live Bacterial Vectors,” International Immunopharmacology 134 (June 2024): 112204. 38703567 10.1016/j.intimp.2024.112204

[19] M. H. Schoots , S. J. Gordijn , S. A. Scherjon , H. van Goor , and J. L. Hillebrands , “Oxidative Stress in Placental Pathology,” Placenta 69 (September 2018): 153–161. 29622278 10.1016/j.placenta.2018.03.003

[20] R. S. Moosavi‐Kohnehsari , M. Jafari‐Sohi , T. Piri‐Gharaghie , S. Tolou‐Shikhzadeh‐Yazdi , M. Aghassizadeh‐Sherbaf , and R. Hosseinzadeh , “A New Vaccination Approach for Salmonellosis Employing a Multi‐Epitope Vaccine Based on Live Microbial Cell Factory From *Lactococcus lactis* ,” Poultry Science 104, no. 2 (February 2025): 104789. 10.1016/j.psj.2025.104789 PMC11954916 39862487

[21] D. Miller , M. Brinkworth , and D. Iles , “Paternal DNA Packaging in Spermatozoa: More Than the Sum of Its Parts? DNA, Histones, Protamines and Epigenetics,” Reproduction 139, no. 2 (February 2010): 287–301. 19759174 10.1530/REP-09-0281

[22] E. Tvrdá , F. Benko , T. Slanina , and S. S. du Plessis , “The Role of Selected Natural Biomolecules in Sperm Production and Functionality,” Molecules 26, no. 17 (August 2021): 5196. 34500629 10.3390/molecules26175196 PMC8434568

[23] A. Kaveh‐Samani , S. Dalali , F. Kaviani , T. Piri‐Gharaghie , and A. Doosti , “Oral Administration of DNA Alginate Nanovaccine Induced Immune‐Protection Against *Helicobacter pylori* in Balb/C Mice,” BMC Immunology 25, no. 1 (February 2024): 11. 38310250 10.1186/s12865-024-00602-6 PMC10838413

[24] S. C. Esteves , D. Santi , and M. Simoni , “An Update on Clinical and Surgical Interventions to Reduce Sperm DNA Fragmentation in Infertile Men,” Andrology 8, no. 1 (January 2020): 53–81. 31692293 10.1111/andr.12724

[25] M. D. Esmatabadi , A. Bozorgmehr , S. N. Hajjari , A. S. Sombolestani , Z. V. Malekshahi , and M. Sadeghizadeh , “Review of New Insights Into Antimicrobial Agents,” Cellular and Molecular Biology 63, no. 2 (February 2017): 40–48. 10.14715/cmb/2017.63.2.6 28364794

