# Supplementary material for: Efficacy and Safety of Hirudotherapy for Improving Sperm Quality Parameters in Male Infertility: A Randomized Controlled Trial
Source: Health Sci Rep. 2026 Feb 23;9(2):e71835. doi: 10.1002/hsr2.71835 (PMC12929192; doi:10.1002/hsr2.71835)
Supplement: Supplementary file 1 — Figure S1: Leech therapy was performed on the experimental group patients. Table S1: The adjusted post‐test averages for the control and experimental groups following leech treatment. Table S2: The T‐test was used to determine if there was a significant difference in the quantity of DNA damage in the experimental group. [file HSR2-9-e71835-s001.docx]

**Supplementary materials**

**Fig S1.** Leech therapy was performed on the experimental group patients


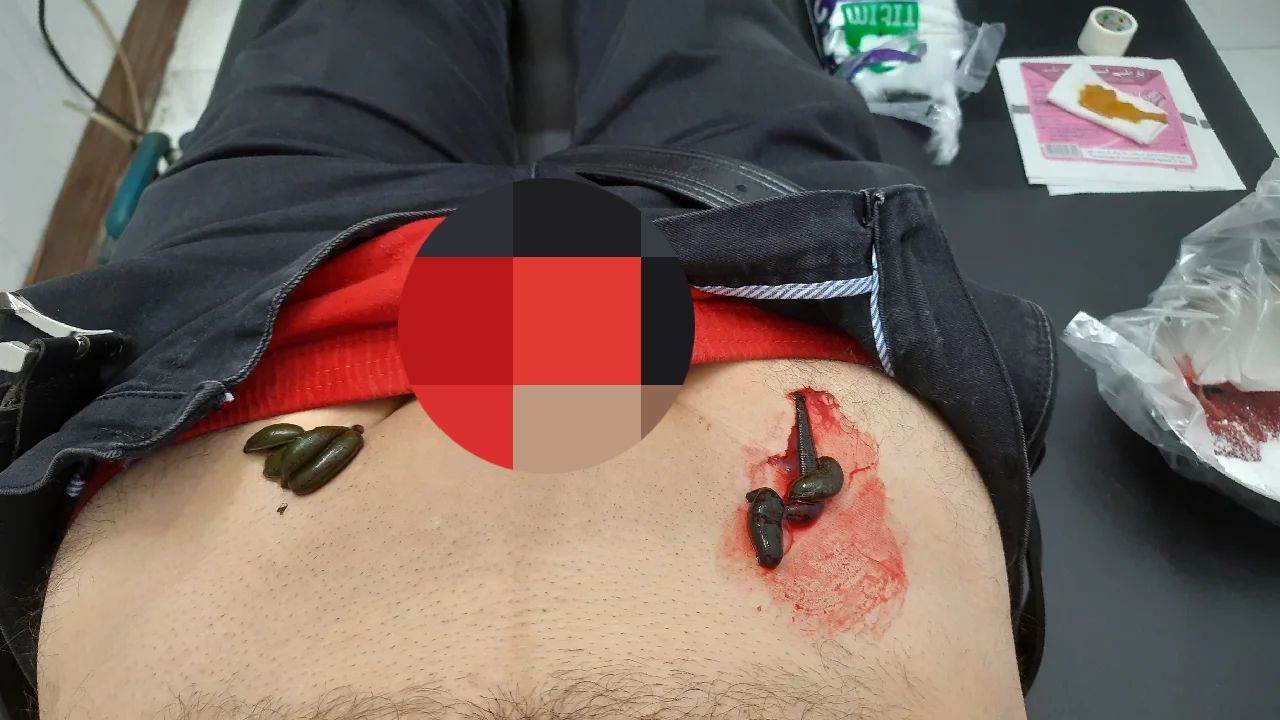


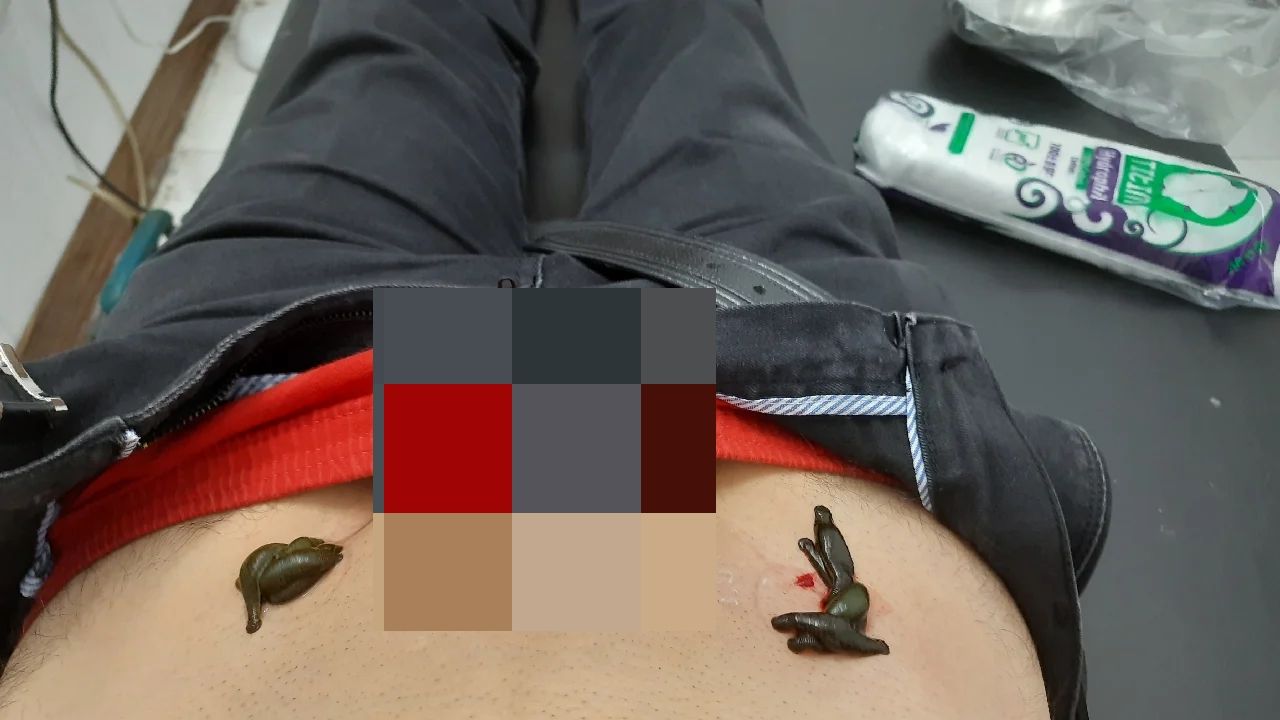


**The effect of leech therapy on reducing the amount of DNA damage in sperm nuclei (DFI = DNA Fragmentation Index)**

The TUNEL test and the Sperm Chromatin Structure test (SCSA) are distinct techniques for evaluating DNA damage in spermatozoa. The TUNEL test has superior accuracy and detection capabilities in recognizing genuine DNA damage, especially in the analysis of double-strand breaks. This test effectively evaluates the degree of DNA damage. The SCSA test utilizes flow cytometry, enabling the analysis of a substantial quantity of sperm in a reduced timeframe while thoroughly evaluating chromatin integrity. The outcomes of this investigation were assessed using covariance analysis and independent t-tests. Table S2 presents the results of the F test conducted to assess the effects between participants, which investigated the study hypothesis about the efficacy of leech treatment in mitigating sperm nuclear DNA damage. The test findings indicated a statistically significant difference in the average sperm nuclear DNA damage between the experimental and control groups (p<0.05). Consequently, the groups exhibited a statistically significant difference from one another. A notable disparity existed between the experimental and control groups post-leech treatment for sperm nuclear DNA damage (p=0.00), with pre-therapy averages being reduced. This validated the beneficial impact of leech treatment on reducing sperm nuclear DNA damage. Table S3 presents the corrected mean of sperm parameters after treatment (post-leech therapy) for the control and experimental groups. This table indicates that the influence of the pre-treatment variable (before leech therapy) has been statistically eradicated.

**TABLE S1 |** The adjusted post-test averages for the control and experimental groups following leech treatment.

| **Dependent variable** | **Group comparison** | **Mean difference** | **Standard deviation** | **Significant value** | **95% confidence interval** | |
| --- | --- | --- | --- | --- | --- | --- |
|  |  |  |  |  | **lower limit** | **upper limit** |
| **The extent of DNA damage** | Experimental -control | -3.9 | 1.8 | 0.04 | -7.7 | -0.14 |

Following the analysis of covariance, the comparative test between the experimental and control groups was also conducted. Table 8 indicated that the disparity in protamine levels and DNA damage scores between the experimental and control groups was 2.7 and 4.9, respectively, which is statistically significant (p<0.05). Table S4 presents the findings of the research on DNA damage reduction as determined by the independent t-test, aligning with the results of the analysis of covariance. In all assays (SCSA and TUNEL), DNA damage was considerably (p<0.05) reduced after leech treatment.

**TABLE S2|** The T-test was used to determine if there was a significant difference in the quantity of DNA damage in the experimental group.

| Test | Groups | Mean ± standard error | Sig. |
| --- | --- | --- | --- |
| SCSA | **Control group** | 1.55±21.43 | P = 0.019 |
|  | **Experimental group** | 1.05±16.9 |  |
| TUNEL | **Control group** | 1.49±16.09 | P = 0.040 |
|  | **Experimental group** | 1.01±12.29 |  |

Leech treatment may enhance sperm parameters and diminish DNA damage in sperm nuclei. The concentration components, total sperm count, progressive motility, complete motility, and normal morphology exhibited a substantial increase in the post-test phase (after leech treatment) compared to the pre-test phase (before leech therapy) (P<0.05). The elements of non-progressive motility, including immotile sperm, abnormal morphology, head defects, neck defects, tail defects, immature cells, protamine levels, and DNA damage levels in the post-test phase (following leech therapy) exhibited a significant reduction compared to the pre-test phase (prior to leech therapy) (P<0.05).

**CONSORT Flow:**

- **Enrollment:
  • 65 men assessed for eligibility
  • 15 excluded (not meeting inclusion criteria / declined to participate)
  • 50 randomized**
- **Allocation:
  • Intervention (hirudotherapy): n = 25 (received allocated intervention: n = 25)
  • Control: n = 25 (received allocated intervention: n = 25)**
- **Follow-Up:
  • Intervention: 0 lost to follow-up, 0 discontinued
  • Control: 0 lost to follow-up, 0 discontinued**
- **Analysis:
  • Intervention: n = 25 analyzed
  • Control: n = 25 analyzed**


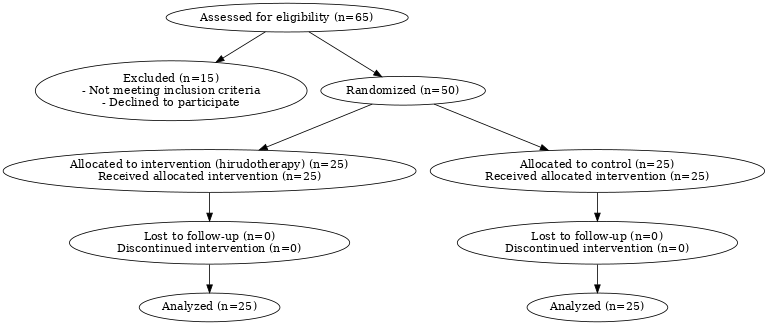


**Figure 1. CONSORT flow diagram showing the progress of participants through the phases of the randomized controlled trial of hirudotherapy for male infertility, including enrollment, allocation, follow-up, and analysis**
